# Eliciting Polyphenols in Strawberry Leaves: Preliminary Experiments in *Fragaria* × *ananassa* cv. Festival

**DOI:** 10.3390/molecules29112467

**Published:** 2024-05-24

**Authors:** Karla Salas-Arias, Andrea Irías-Mata, Laura Sánchez-Calvo, María Fernanda Brenes-Zárate, Ana Abdelnour-Esquivel, Fabián Villalta-Romero, Laura A. Calvo-Castro

**Affiliations:** 1 Doctorado en Ciencias Naturales para el Desarrollo (DOCINADE), Instituto Tecnológico de Costa Rica, Universidad Nacional, Universidad Estatal a Distancia, Cartago P.O. Box 159-7050, Costa Rica; ksalas@itcr.ac.cr; 2 Escuela de Biología, Instituto Tecnológico de Costa Rica, Cartago P.O. Box 159-7050, Costa Rica; mbreneszarate@gmail.com (M.F.B.-Z.); aabdelnour@gmail.com (A.A.-E.); fvillalta@itcr.ac.cr (F.V.-R.); 3 Centro para Investigaciones en Granos y Semillas, Escuela de Agronomía, Universidad de Costa Rica, San José P.O. Box 2060, Costa Rica; andrea.iriasmata@ucr.ac.cr; 4 Escuela de Ciencias Exactas y Naturales, Universidad Estatal a Distancia, San José P.O. Box 474-2050, Costa Rica; lsanchezc@uned.ac.cr

**Keywords:** elicitors, leaves, polyphenols, strawberry

## Abstract

Polyphenols are plant secondary metabolites that function mostly as a general stress-induced protective mechanism. Polyphenols have also gained interest due to their beneficial properties for human health. Strawberry leaves represent an agro-industrial waste material with relevant bioactive polyphenol content, which could be incorporated into circular economy strategies. However, due to the low quantities of polyphenols in plants, their production needs to be improved for cost-effective applications. The objective of this research was to compare polyphenol production in strawberry (*Fragaria* × *ananassa* cv. Festival) leaves in plants grown in greenhouse conditions and plants grown in vitro, using three possible elicitor treatments (UV irradiation, cold exposure, and cysteine). General vegetative effects were morphologically evaluated, and specific polyphenolic compounds were quantified by UHPLC-DAD-MS/MS. Gallic acid was the most abundant polyphenol found in the leaves, both in vivo and in vitro. The results showed higher amounts and faster accumulation of polyphenols in the in vitro regenerated plants, highlighting the relevance of in vitro tissue culture strategies for producing compounds such as polyphenols in this species and cultivar.

## 1. Introduction

Polyphenols are plant secondary metabolites involved in plant growth and development processes, and they can accumulate in plants in response to several environmental biotic and abiotic stressors [1]. Polyphenols are also interesting for their capacities to regulate human metabolism and to promote human health [2,3,4]. However, plant secondary metabolites account for less than 1% of the dry weight of plants, which fails to satisfy commercial demand [5]. Moreover, the industrial production of polyphenols has been limited by their difficult extraction and costly synthesis, due to their complex structures characterized by the union of several phenolic groups with very specific stereochemistry and variable polarity [6,7,8].

Strawberries (*Fragaria* × *ananassa*) are a fruit available worldwide with a relevant polyphenol content [9,10] and known bioactive properties [11,12]. From a global production of over 12 million tons per year, around 20% of strawberry production corresponds to vegetative waste, which is generally discarded [13,14]. Nonetheless, strawberry leaves have been found to exhibit higher polyphenol content and higher antioxidant activity than strawberry fruits, representing a presumably relevant source of unexploited bioactive biomass [15]. Furthermore, as a temperate crop, tropical highland strawberry crops have been less studied, even though increased polyphenol accumulation and diversity has been shown to occur in other crops under the higher temperatures, longer photoperiods, and the increased UV irradiation conditions of lower latitudes [16].

The quantity and quality of polyphenols in plants depend on several factors such as genetics, growth conditions (light, temperature, humidity, soil, salinity), physiological maturity, processing, and storage [1,17]. Several biotechnological production systems have been tested for enhancing plant metabolite yield, including the selection of high-yielding lines; plant cell, tissue, and organ culture; precursor feeding; elicitation; large-scale cultivation in bioreactor systems; genetic engineering; and biotransformation [5,18,19,20]. Several strategies have been tested on strawberry crops to enhance plant growth and quality (reviewed in [21]). However, most studies have focused on the fruit.

In addition to collecting strawberry leaves at the end of each crop cycle, and to avoid harming the quality and yield of strawberry fruits (the economically and nutritionally relevant product), vegetative polyphenol production could be exploited and even enhanced via in vitro plant and tissue culture systems, together with the use of exogenous biotic or abiotic elicitors to induce plant secondary metabolism responses, which may result in increased production or in new forms of bioactive compounds [5,19,22,23]. Previous studies in strawberry crops have achieved enhanced leaf size and number and increased phenolics and antioxidant capacity using elicitors such as humic substances, protein hydrolysates, microbial supplementation (microalgae, bacteria, fungi), plant extracts, biopolymers (e.g., chitosan), minerals, diverse nanoparticle and compost formulations, and abiotic stimulants such as ultraviolet (UV) and visible light, heat, cold, and magnetic field exposure (reviewed in [21]).

For this study, we conducted a preliminary evaluation of polyphenol production in strawberry (*F.* × *ananassa* cv. Festival) leaves from plants regenerated in vitro with three easy-to-implement and inexpensive elicitor treatments (UV irradiation, low overnight temperatures, and foliar cysteine), and compared their polyphenol content with those of leaves from plants grown in greenhouse conditions in a tropical highland environment. To the best of our knowledge, this is the first report showing polyphenol elicitation in the leaves of the strawberry cv. Festival from Costa Rica.

## 2. Results and Discussion

To establish the plants in vitro, two disinfection protocols were applied to the strawberries (*Fragaria* × *ananassa* cv. Festival) apical and axillary runners (*n* = 24, each) from greenhouse plants. Disinfectant Gamba Oxi was more effective (81.67 ± 15.04% and 91.00 ± 2.00% apical and axillary runner success, respectively) for the elimination of contaminants than treatment with chlorine (68.33 ± 15.95% and 65.00 ± 6.08% apical and axillary runner success, respectively). Overall, success rates were very similar between apical (75.00 ± 15.67%) and axillary (78.00 ± 14.81%) explants. Micropropagation of the established in vitro strawberry plants in MS medium supplemented with 0.5, 1, and 1.5 mg/L BAP showed sprouting with roots in all concentrations after 6 weeks. However, in vitro plants with 0.5 mg L^−1^ BAP had slightly better qualitative morphological development, and subsequent culture was continued only with this formulation. After 12 weeks, two generations (P1–P2) were produced, corresponding to 105 in vitro plants with a yield percentage of 95.8%.

Total polyphenol content (mg GAE g^−1^ of dry weight) in the in vitro and in vivo strawberry plants (*F.* × *ananassa* cv. Festival) treated with UV irradiation, cold exposure, and cysteine for 15 days was very similar to their respective untreated controls (Table 1). However, total polyphenol content was about two times higher in the in vitro plants relative to the greenhouse-grown seedlings. Increased or differential biosynthesis and accumulation of secondary metabolites in the in vitro regenerated plants relative to their in vivo or ex vitro counterparts has been reported in other plants (reviewed in [24]) highlighting the relevance of this biotechnological strategy for producing compounds such as polyphenols in higher amounts and more quickly than in conventional crops.

Although total polyphenol production was apparently not affected by the elicitor treatments, there were differences in the specific polyphenols detected and quantified by UHPLC-MS/MS-DAD. Six polyphenols were identified and quantified in the leaves from both in vivo and in vitro plants (gallic acid, caffeic acid, chlorogenic acid, *p*-coumaric acid, ellagic acid, and quercetin) (Table 2 and Table 3). Two other polyphenols, rutin and (+)-catechin, were present only in the in vivo (in trace amounts) or in the in vitro plants, respectively. As reported before for this cultivar [15], gallic acid was the most abundant polyphenol found in the leaves, both in vivo (in the insoluble fraction) and in vitro, under most treatments (except for the cysteine-treated in vivo plants, where rutin was the more abundant polyphenol). Moreover, in accordance with the total polyphenol results, gallic acid concentrations were also higher in the in vitro plants (Table 2) relative to the greenhouse-grown seedlings (Table 3).

Of note, chlorogenic acid was found only in elicitor-treated plants, and quercetin was present in vitro only in the cysteine-treated plants, in concentrations 15 times higher than in the untreated control. (+)-Catechin and chlorogenic acid were also absent in untreated greenhouse-grown strawberry (*F.* × *ananassa* cv. Festival) leaves according to a previous report, where quercetin was also found in much lower concentrations [15]. These three compounds warrant further investigation in the future as possible targets for polyphenol elicitation in strawberry leaves. Kotsupiy et al. [25] also reported higher phenolic acids concentrations in strawberry (*F.* × *ananassa* cv. Solnechnaya polyanka) plants in vitro, and higher quercetin and (+)-catechin content in greenhouse-acclimated plants. These authors suggest that a reduced stress load favors the accumulation of phenolic acids in vitro, and that they are transformed into cell-wall-bound species during greenhouse growth due to lignification and increased stress conditions. Accordingly, all polyphenols quantified in vivo in our study were present in much higher concentrations in the insoluble fraction, suggesting that the polyphenols detected in the strawberry leaves consisted mostly of insoluble cell-wall-bound phenolics, with only very low amounts of soluble species.

Finally, we also conducted a morphological characterization of the plants to evaluate the effect of the elicitors in general plant growth and development. In vitro, the UVC and the cold treatments induced significantly (*p* < 0.05) longer leaf lengths relative to the untreated control (Table 4); UVC also caused significantly more leaves and buds. In vivo, only the length of roots and stems were significantly increased by the elicitors, and only by exposure to low temperatures (4 °C) or to foliar cysteine (200 mg L^−1^) (Table 5). However, none of the treatments increased plant biomass relative to their respective controls, in vivo or in vitro. These results suggest that normal plant growth was not impaired by the treatments in the short term. However, considering that the economically relevant product is the strawberry fruit, future studies should evaluate the effect of these treatments on crop yield and quality in mature plants.

**Figure 1:**
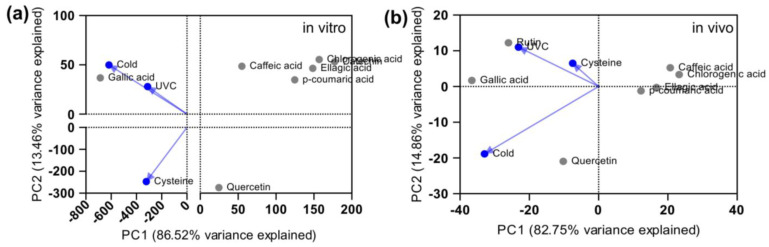
Principal component analysis (PCA) of specific polyphenol content in strawberry (*Fragaria* × *anannasa* cv. Festival) leaves from plants grown in vitro (**a**) and in vivo (**b**) after treatment with three elicitors (UV irradiation, cold exposure, and foliar cysteine) for 15 days (GraphPad Prism v. 10.1.1., USA).

Due to the small sample size, it was not possible to statistically compare the differences between the elicitor treatments; however, a principal component analysis (PCA) (Figure 1) suggests that gallic acid was the polyphenol most influenced by the elicitor treatments, amongst which the cold treatment had the greater weight. Cold tolerance is an intrinsic characteristic of *F.* × *ananassa,* a perennial plant, which is mostly grown in temperate climate zones, freezing in the winter and re-growing in spring [26]. Cold stress has been shown to induce polyphenol synthesis as antioxidants in strawberry fruits and other plants [27]. Given that the cold treatment induced large leaf size in vitro, which could translate to larger biomass, and considering that tropical highland strawberry crops rarely freeze, future experiments should consider the relevance of stimulating cold-regulated responses in the shikimate pathway for polyphenol accumulation, particularly using in vitro plant production systems.

To the best of our knowledge, this is the first report comparing the polyphenol profile of *F.* × *ananassa* cv. Festival leaves in plants grown in a tropical highland environment under three polyphenol-eliciting treatments in vitro vs. their counterparts in vivo, showing higher amounts and faster accumulation of polyphenols in the in vitro regenerated plants. This information should be considered in further experiments using other biotechnological strategies, such as large-scale cultivation in bioreactor systems.

## 3. Materials and Methods

Reagents and chemicals. The sodium hypochlorite (3%), citric acid (>99% purity), and ascorbic acid (99% purity) were purchased from LABQUIM CR (Alajuela, Costa Rica). The Gamba Oxi^®^ (2% hydrogen peroxide, 1% peracetic acid, 2% acetic acid) was purchased from RACKAM S.A. (Alajuela, Costa Rica). The Agri-mycin^®^ 16.5 WP (oxytetracycline + streptomycin sulfate) was from Pfizer S.A. (México-Toluca, México). The Afungil^®^ 50 WP (benomyl) was from SERACSA (Cartago, Costa Rica). The 6-benzylaminopurine (99% purity) and Murashige & Skoog macro- and micronutrient salt bases were obtained from PhytoTech Labs (Lenexa, KS, USA). The L-cysteine, Folin & Ciocalteu’s phenol reagent and gallic acid, (+)-catechin, caffeic acid, chlorogenic acid, *p*-coumaric acid, ellagic acid, quercetin, and rutin (all standards > 99% purity) were obtained from Sigma-Aldrich (St. Louis, MO, USA). The methanol and ethyl acetate (>99% HPLC-grade) were from Honeywell Riedel-de-HaënTM (Muskegon, MI, USA).

Plant material. The strawberry (*F.* × *ananassa* cv. Festival) plants were obtained from Fresas de Altura S.A. in Llano Grande, Cartago, Costa Rica (permit R-CM-ITCR-001-2022-OT-CONAGEBIO), they were kept in a greenhouse at the Costa Rica Institute of Technology (Cartago, Costa Rica) under natural temperature (17–25 °C) and light (~12 h daylight) conditions (August to December 2021), and they were watered daily in the morning and in the afternoon.

In vitro culture. The introduction and multiplication were based on the protocols by Jiménez-Bonilla & Abdelnour-Esquivel [28]. The explants introduced in vitro consisted of runners with apical meristems and runners with axillary meristems (approximately 3 cm long). For disinfection, explants (*n* = 24, each) were treated with surgical soap, water, Agri-mycin^®^ + Afungil^®^, and a final step of sodium hypochlorite (treatment A) or Gamba Oxi (treatment B) (Table 6). After disinfection, the explants were rinsed three times with sterile distilled water in aseptic conditions and kept in an antioxidant solution of sterile citric acid (300 mg L^−1^) and ascorbic acid (300 mg L^−1^) while they were introduced in cylindrical test tubes containing 10 mL of a complete standard Murashige & Skoog (MS) medium [29] without growth regulators. The pH was adjusted to 5.7 with NaOH 1 M. The explants were incubated in a clean room for 8 days at 27 °C in darkness, followed by light exposure (with a photoperiod of 16 light hours at 2000 lux and 8 h in diffuse light) for 4 weeks. Contamination was evaluated every week. The aseptically established plants were subsequently grown in complete MS medium with 6-benzylaminopurine (BAP) in different concentrations (0.5, 1, and 1.5 mg L^−1^) and subcultivated every 6 weeks.

Elicitor treatments. Three elicitor treatments (UV irradiation, cold exposure, and cysteine) were tested in strawberry (*F.* × *ananassa* cv. Festival) seedlings kept in a greenhouse or in in vitro plants. For the cold treatment, the plants were incubated at 4 °C for 12 h daily in the dark. The UV treatment consisted of irradiating the plants with 254 nm (UV-C) for 7 min each day at the same hour. Finally, 7 mL foliar cysteine (200 mg L^−1^) was applied daily over the leaves of each seedling, or it was added once to the semisolid medium (in vitro plants) at the same concentration. These treatments were established from preliminary tests, based on practicality, local availability, and cost. The seedlings were exposed to each of the three elicitors for 15 days (in vivo and in vitro) to evaluate their individual effects. However, due to minor changes in plant morphology, a second experiment was conducted exposing the plants to all elicitors combined for 30 days (in vivo). The morphological features were measured in all plants at the end of the experiments.

Polyphenol extraction. All the leaves from the plants in vivo and in vitro were freeze-dried and pulverized in a food processor. Insoluble (cell-wall-bound) and soluble (free, soluble ester- and glycoside-bound) polyphenols were extracted using aqueous methanol and ethyl acetate, respectively, as described before [30,31]. In the in vitro plants, due to low biomass, the dried leaves had to be pooled together in three groups of 10 replicates each (*n* = 3), resulting in a single control sample; and the dried material was only solubilized in methanol (10 mg mL^−1^) before the chromatographic analysis. 

Total polyphenol content (TPC). TPC was determined in the water–ethanol extract (in vivo plants) or in the methanol-solubilized leaves (in vitro plants) by the Folin–Ciocalteu method [32]. TPC was calculated by comparing with an external calibration curve of gallic acid (10–80 mg L^−1^, *r*^2^ = 0.9863) (>99% purity, Sigma-Aldrich, St. Louis, MO, USA).

Chromatographic analysis. The methanol-dissolved samples were analyzed in an ultra-high-performance liquid chromatography system (UHPLC) with a triple quadrupole mass spectrometer (MS/MS) and a diode-array detector (DAD, Ultimate 3000, TSQ Endura, series TQH-E1-0288, Thermo Fisher Scientific, Waltham, MA, USA), as previously reported [31,33]. Mass detection of polyphenols was carried out with an electrospray ionization (ESI) source operating in negative polarity with SRM scan mode with a cycle time of one second. Mass settings were as follows: spray voltage, 2500 V (negative); sheath gas flow, 50 arb; auxiliary gas flow, 10 arb; sweep gas flow, 1 arb; ion transfer tube temperature, 300 °C; vaporizer temperature, 350 °C; and collision induced gas, 1.5 mTorr. Peaks were analyzed with the Chromeleon software (version 7.3, Thermofisher Scientific, Waltham, MA, USA) and identified and quantified by UV–vis wavelengths and mass spectra using the external curves of authentic polyphenol standards (Sigma-Aldrich, St. Louis, MO, USA). At least four samples were analyzed per treatment and each one was injected once for UHPLC-MS/MS-DAD analysis.

Statistical analysis. Normality was tested and the differences between the untreated controls and the elicitor treatments were calculated with a two-tailed unpaired *t* test, a one-way analysis of variance (ANOVA) or with a Kruskal–Wallis test, with the corresponding post hoc tests. A principal component analysis (center method; principal components selected based on eigenvalues greater than 1) was conducted to evaluate relationships between the elicitor treatments and the polyphenol content. All statistical analyses were performed with Graph-Pad Prism (v. 10.1.1., GraphPad Software, San Diego, CA, USA).

## Figures and Tables

**Table 1:** Total polyphenol content (mean ± SD) determined by the Folin–Ciocalteu method in strawberry (*F.* × *ananassa* cv. Festival) leaves after 15 days of treatment with three elicitors (UV irradiation, cold exposure, and cysteine) in vitro (*n* = 3, except for control) and in vivo (*n* = 6).

Treatment	Total Polyphenols (mg GAE g^−1^ DW)	
	In Vitro	In Vivo
Untreated control	212.27 *	116.18 ± 17.06
UVC (254 nm)	250.45 ± 119.94	117.54 ± 13.94
Cold (4 °C)	203.33 ± 35.09	112.77 ± 19.12
Cysteine (200 mg L^−1^)	215.90 ± 81.30	110.54 ± 16.75

DW, dry weight. * Due to low biomass, all ten replicates had to be pooled together for analysis. There were no statistical differences (*p* < 0.05) between treatments in vivo (two-tailed unpaired *t* test; GraphPad Prism v. 10.1.1., USA).

**Table 2:** Specific polyphenols (mean ± SEM; *n* = 3) in strawberry (*Fragaria* × *ananassa* cv. Festival) leaves from in vitro plants determined by UHPLC-DAD-MS/MS after 15 days of treatment with three elicitors (UVC irradiation, overnight 4 °C exposure, and 200 mg L^−1^ cysteine).

Peak	Compound	Retention Time (min)	UV/Vis Wavelength (nm)	[M–H]:Ion Products (*m*/*z*)	Polyphenol Concentration in µg mg^−1^ DW			
					Control	UVC (254 nm)	Cold (4 °C)	Cysteine (200 mg L^−1^)
1	Gallic acid	2.06	201/220/270	169:79, 81, 125	132.48	355.34 ± 69.43	690.03 ± 250.47	379.62 ± 38.53
2	(+)-Catechin	7.13	201/278	289:203, 205, 245	0.0019	0.003 ± 0.0006	0.002 ± 0.0003	0.002 ± 0.0001
3	Caffeic acid	8.51	216/240/322	179:107, 117, 135	9.99	49.95 ± 9.77	96.93 ± 31.47	55.81 ± 12.20
4	Chlorogenic acid	8.69	215/320	353:135, 179, 191	n.d.	15.05 ± 1.36	14.18 ± 0.81	6.66 ± 1.39
5	*p*-coumaric acid	10.51	209/310	163:93, 117, 119	11.30	26.13 ± 4.39	31.53 ± 14.87	39.00 ± 14.02
7	Ellagic acid	11.15	196/254/366	301:185, 229, 257	11.39	22.58 ± 5.56	14.66 ± 9.25	18.45 ± 2.84
8	Quercetin	13.91	201/255/370	301:121, 151	23.82	n.d.	n.d.	361.79 ± 54.98

n.d. = not detected. Peak numer 6 (rutin) was not detected in these samples. Due to low biomass, the dried leaves were pooled into three groups of ten replicates each, yielding only one pooled control sample. There were no significant differences between elicitor treatments at *p* < 0.05 (Kruskal–Wallis test followed by Dunn’s multiple comparison test within each row; GraphPad Prism v. 10.1.1., USA).

**Table 3:** Specific polyphenols (mean ± SEM; *n* = 4) in soluble (free) and insoluble (bound) polyphenol fractions of strawberry (*Fragaria* × *ananassa* cv. Festival) leaves from greenhouse-grown seedlings determined by UHPLC-DAD-MS/MS after 15 days of treatment with three separate elicitors (UVC irradiation at 254 nm, overnight 4 °C exposure, and 200 mg L^−1^ foliar cysteine).

Peak	Compound	Retention Time (min)	UV/Vis Wavelength (nm)	[M–H]:Ion Products (*m*/*z*)	Polyphenol Concentration in µg mg^−1^ DW Extract					
					Insoluble Fraction			Soluble Fraction		
					UVC	Cold	Cysteine	UVC	Cold	Cysteine
1	Gallic acid	2.06	201/220/270	169:79, 81, 125	35.01 ± 6.74	51.17 ± 11.49	5.47 ± 0.72	0.58 ± 0.26	0.35 ± 0.12	0.26 ± 0.03
3	Caffeic acid	8.51	216/240/322	179:107, 117, 135	2.41 ± 0.58	3.66 ± 0.56	2.03 ± 0.56	0.36 ± 0.27	0.37 ± 0.08	3.66 ± 0.56
4	Chlorogenic acid	8.69	215/320	353:135, 179, 191	n.d.	2.61 ± 0.75	n.d.	1.30 ± 0.66	0.51 ± 0.15	0.82 ± 0.22
5	*p*-coumaric acid	10.51	209/310	163:93, 117, 119	2.31 ± 0.23	14.32 ± 6.33	1.61 ± 0.08	0.66 ± 0.01	0.86 ± 0.36	0.35 ± 0.17
6	Rutin	10.63	201/256/355	609:151, 255, 271, 300, 301	30.52 ± 3.02	37.92 ± 14.13	19.87 ± 1.30	4.36 ± 0.67	1.89 ± 0.65	3.52 ± 0.43
7	Ellagic acid	11.15	196/254/366	301:185, 229, 257	0.65 ± 0.29	10.12 ± 4.50	0.76 ± 0.40	0.01 ± 0.01	0.19 ± 0.19	0.001 ± 0.001
8	Quercetin	13.91	201/255/370	301:121, 151	n.d.	44.19 ± 9.37	n.d.	0.13 ± 0.01	0.16 ± 0.01	0.12 ± 0.01

n.d. = not detected. Peak numer 2 ((+)-catechin) was not detected in these samples. There were no significant differences between elicitor treatments at *p* < 0.05. (Kruskal–Wallis test followed by Dunn’s multiple comparison test within each row; GraphPad Prism v. 10.1.1., USA).

**Table 4:** Morphological characterization (mean ± SD) of strawberry plants (*F.* × *ananassa* cv. Festival) in vitro (*n* = 30, except for control with *n* = 10) after treatment with three elicitors (UV irradiation, cold exposure, and cysteine) for 15 days.

Parameter	Control	UVC (254 nm)	Cold (4 °C)	Cysteine (200 mg L^−1^)
Length of the longest root (cm)	0.89 ± 0.93	1.49 ± 0.90	1.39 ± 0.48	1.33 ± 0.81
Length of the longest stem (cm)	2.14 ± 0.66	2.93 ± 0.99	2.66 ± 1.01	2.49 ± 0.89
Length of the longest leaf (cm)	0.94 ± 0.25	1.23 ± 0.26 *	1.21 ± 0.30 *	1.10 ± 0.32
Width of the longest leaf (cm)	0.99 ± 0.23	1.18 ± 0.29	1.06 ± 0.31	1.15 ± 0.33
Total amount of leaves	19.50 ± 5.10	38.53 ± 24.9 *	25.93 ± 9.22	21.30 ± 4.49
Total amount of buds	3.00 ± 0.82	5.47 ± 2.74 *	3.87 ± 1.76	4.47 ± 1.41
Fresh weight of leaves (g)	0.55 ± 0.28	0.59 ± 0.42	0.39 ± 0.37	0.36 ± 0.24

Differences (*p* < 0.05) in treatments relative to their respective control were analyzed by one-way ANOVA followed by Dunnett’s test (GraphPad Prism v. 10.1.1., USA) and are marked by asterisks.

**Table 5:** Morphological characterization (mean ± SD) of strawberry (*F.* × *ananassa* cv. Festival) greenhouse-grown seedlings (*n* = 6) after treatment with three elicitors (UV irradiation, cold exposure, and foliar cysteine) for 15 days.

Parameter	Control	UVC (254 nm)	Cold (4 °C)	Cysteine (200 mg L^−1^)
Length of the longest root (cm)	11.30 ± 1.47	10.78 ± 1.63	14.08 ± 2.66 *	13.68 ± 0.85
Length of the longest stem (cm)	7.28 ± 0.54	8.96 ± 1.35	9.52 ± 0.39 *	9.07 ± 1.15 *
Length of the longest leaf (cm)	3.47 ± 0.37	3.20 ± 0.49	3.63 ± 0.16	3.50 ± 0.62
Width of the longest leaf (cm)	3.03 ± 0.20	2.78 ± 0.37	3.20 ± 0.20	2.95 ± 0.36
Total amount of leaves	12.67 ± 3.14	13.50 ± 1.64	14.33 ± 3.50	16.00 ± 4.10
Total amount of buds	4.33 ± 1.21	5.17 ± 0.75	5.33 ± 1.21	5.83 ± 2.04
Fresh weight of leaves (g)	1.08 ± 0.21	0.98 ± 0.23	1.37 ± 0.41	1.35 ± 0.59

Differences (*p* < 0.05) in treatments relative to their respective control were analyzed by one-way ANOVA followed by Dunnett’s test (at 15 days) (GraphPad Prism v. 10.1.1., USA) and are marked by asterisks.

**Table 6:** Disinfection protocol of strawberry explants for in vitro culture.

Treatment	Disinfectant Steps	Working Concentration	Incubation Time in Agitation (min)
**A**	Surgical soap	-	10
	Water	-	5
	Agri-mycin^®^ + Afungil^®^	5 g L^−1^, 4 g L^−1^	15
	NaClO	3%	15
**B**	Surgical soap	-	10
	Water	-	5
	Agri-mycin^®^ + Afungil^®^	0.8 g L^−1^, 0.8 g L^−1^	15
	Gamba Oxi	40%	5

## Data Availability

Datasets are available from the corresponding author on reasonable request.
